# Stability Enhancement of a Dimeric HER2-Specific Affibody Molecule through Sortase A-Catalyzed Head-to-Tail Cyclization

**DOI:** 10.3390/molecules26102874

**Published:** 2021-05-12

**Authors:** Kristina Westerlund, Anders Myrhammar, Hanna Tano, Maxime Gestin, Amelie Eriksson Karlström

**Affiliations:** Department of Protein Science, School of Engineering Sciences in Chemistry, Biotechnology and Health, KTH Royal Institute of Technology, AlbaNova University Center, SE-10691 Stockholm, Sweden; krw@kth.se (K.W.); andersn8@kth.se (A.M.); htano@kth.se (H.T.); gestin@kth.se (M.G.)

**Keywords:** cyclization, thermal stability, proteolytic stability, sortase A, Affibody

## Abstract

Natural backbone-cyclized proteins have an increased thermostability and resistance towards proteases, characteristics that have sparked interest in head-to-tail cyclization as a method to stability-enhance proteins used in diagnostics and therapeutic applications, for example. In this proof-of principle study, we have produced and investigated a head-to-tail cyclized and HER2-specific Z_HER2:342_ Affibody dimer. The sortase A-mediated cyclization reaction is highly efficient (>95%) under optimized conditions, and renders a cyclic Z_HER3:342_-dimer with an apparent melting temperature, T_m_, of 68 °C, which is 3 °C higher than that of its linear counterpart. Circular dichroism spectra of the linear and cyclic dimers looked very similar in the far-UV range, both before and after thermal unfolding to 90 °C, which suggests that cyclization does not negatively impact the helicity or folding of the cyclic protein. The cyclic dimer had an apparent sub-nanomolar affinity (K_d_ ~750 pM) to the HER2-receptor, which is a ~150-fold reduction in affinity relative to the linear dimer (K_d_ ~5 pM), but the anti-HER2 Affibody dimer remained a high-affinity binder even after cyclization. No apparent difference in proteolytic stability was detected in an endopeptidase degradation assay for the cyclic and linear dimers. In contrast, in an exopeptidase degradation assay, the linear dimer was shown to be completely degraded after 5 min, while the cyclic dimer showed no detectable degradation even after 60 min. We further demonstrate that a site-specifically DyLight 594-labeled cyclic dimer shows specific binding to HER2-overexpressing cells. Taken together, the results presented here demonstrate that head-to-tail cyclization can be an effective strategy to increase the stability of an Affibody dimer.

## 1. Introduction

Affibody molecules are a class of small—58 residues and 7 kDa—non-immunoglobulin affinity proteins that have been selected to bind with high affinity to a wide range of different protein targets such as epidermal growth factor receptor (EGFR) [1], human epidermal growth factor receptor 2 (HER2) [2], human epidermal growth factor receptor 3 (HER3), [3] insulin-like growth factor 1 receptor (IGF1R) [4], and carbonic anhydrase IX (CAIX) [5].

The Affibody scaffold was originally based on a stability enhanced variant of the B-domain, called the Z-domain, from the IgG-binding protein A of *Staphylococcus aureus.* The three-helical bundle and cysteine-free Affibody scaffold has rapid folding kinetics, high solubility, and a relatively high thermal stability, which makes the Affibody technology an attractive research tool for biotechnological and pharmaceutical applications [6,7]. One of the therapeutically relevant Affibody molecules binds to the HER2 receptor, which is associated with an aggressive form of breast cancer. This Affibody molecule has been extensively studied as a tracer for molecular imaging of HER2-overexpressing tumors (e.g., by PET), and as therapeutic agent for the targeted delivery of cytotoxic drugs or radionuclides. When used for therapeutic purposes, the HER2-binding Affibody has often been produced as a dimeric construct, to increase the apparent binding affinity to HER2 [2,8]. The aim of this current study was to stabilize a dimeric anti-HER2-Affibody against proteolytic degradation and to enhance the thermal stability by taking inspiration from natural head-to-tail circulated proteins.

Ribosomally synthesized and backbone-cyclized proteins have been identified in mammals, plants, fungi, and bacteria, and are often involved in the host defense system. Compared to ordinary linear proteins, head-to-tail cyclized proteins are characterized by their often-high thermal stability, resistance towards many proteases, and stability over broad pH ranges [9]. The most well-studied group of cyclic peptides/proteins are the small (~30 residues) and ultra-stable plant cyclotides, which are produced in large quantities by certain plant families in defense against pests and pathogens. Cyclic bacteriocins, antimicrobial proteins produced by Gram-negative bacteria, are another group of backbone-cyclized proteins with exceptional thermal and pH stability. One example is the pore-forming cyclic bacteriocin Enterocin AS-48 from *Enterococcus faecalis* which has a compact and globular five-helix fold. The highly basic AS-48 has one of the highest reported thermal unfolding temperatures reported in the literature, 102 °C at pH 2.5 and low ionic strength [10]. In contrast to many eukaryotic cyclic proteins, AS-48 does not contain intramolecular disulfide bonds, but backbone cyclization is essential for the correct fold because the cyclization site is situated in the middle of a helix [9]. 

The relative simplicity of tying the peptide backbone together, and the potential large gain in stability that can be achieved, has sparked interest in circular proteins for industrial, biopharmaceutical, and biotechnological applications. Head-to-tail cyclization by chemical methods, such as native chemical ligation [11], has proven difficult for larger proteins and can suffer by low yields of the circular product even for shorter peptides [12]. Intein- [13] and enzymatically based methods, i.e., using sortase A [14] and butelase-1 [15], have emerged as efficient ways to generate cyclic peptides and proteins [16]. Sortase A was originally isolated from *Staphylococcus aureus* where it functions to anchor proteins containing an LPXTG-recognition sequence (where X is any amino acid) to penta-glycine cross-bridges in the peptidoglycan cell wall. Recombinantly expressed sortase A has become an important tool for protein engineering, and the enzyme has been used to covalently conjugate proteins to other biomolecules, small synthetic molecules and to surfaces (reviewed in [17,18]). Broder and coworkers were the first group to describe sortase A-mediated backbone-cyclization of a recombinant protein when they incubated a bifunctional GFP-variant, containing both N-terminal glycines and a C-terminal LPETG recognition sequence, with the enzyme [19]. A similar approach was used by Ploegh et al. to cyclize GFP with ≥90% conversion efficiency, and the authors noted that the intra-molecular transpeptidation reaction catalyzed by sortase A is remarkably similar to the cyclization step in the biosynthesis of cyclotides [15]. Since then, sortase A has been used to catalyze head-to-tail cyclization of short SPPS-synthesized peptides [20], cytokines [21], human growth hormone [22], and even to cyclize proteins in vivo in *Saccharomyces cerevisiae* and human HEK293T cells [23].

Intramolecular crosslinking and backbone cyclization of Affibody molecules and Z-domain variants have previously been investigated by our group and others [24,25,26]. An intramolecular thioether bond was, for example, shown to thermally stabilize a solid-phase synthesized and monomeric HER2-binding Affibody by 10 °C [24], and in a chemically synthesized 2-helix variant of the Z-domain, the introduction of a native peptide bond connecting the N-terminus to the C-terminus improved the ability of the protein to refold following thermal denaturation [25].

In this proof-of-principle study, we have produced and investigated a backbone-cyclized Z_HER2:342_-dimer produced using sortase A mediated ligation. We designed a dimeric but single-chain construct with two Z_HER2_-domains connected by a 15-residue linker (–EFGSGSGSCPGSGGG–) containing a unique cysteine residue. The cysteine can be used to label the dimeric constructs with thiol-reactive compounds, such as maleimide-derivatized fluorophores and radiometal chelators, or to immobilize the construct to surfaces or beads. The construct has N-terminal glycines and a C-terminal sortase A recognition sequence, SR, making the dimer a substrate for intramolecular sortase A-catalyzed cyclization. Upon sortase A treatment, this protein construct is designed to form a head-to-tail cyclized 146-residue protein. This dimeric and cyclic Z_HER2:342_ protein was biophysically characterized using MALDI-MS, surface plasmon resonance (SPR), and circular dichroism (CD) and compared to its linear counterpart. To assess whether backbone-cyclization protects the dimer from proteolytic degradation, the linear and cyclic proteins were treated with both exopeptidases and endopeptidases in separate experiments. Labeling of the unique cysteine residue with a DyLight 594 maleimide dye was performed in order to assess the binding of the cyclic protein to HER2-expressing cells by fluorescence microscopy.

## 2. Materials and Methods

### 2.1. Construction of Expression Plasmids, Expression and Purification of Recombinant Proteins

The plasmid coding for the dimeric Z_HER2:342_ protein was made in two steps. Firstly, the DNA sequence of Z_HER2:342_ was amplified from pAY430-Z_HER2:342_-SR-H_6_ [27] using PCR primers that introduced three N-terminal glycines and a C-terminal peptide linker containing a unique cysteine residue followed by BamHI and AccI restriction sites (5′-CCATCCATATGGGCGGTGGCGTAGATAACAAATTCAACAAAGAAATGC-3′ and 5′CGATGGTCTACCGTCCGGATCCCGGGCAAGATCCAGATCCAGAGCCGAATTCTTTCGGCGCCTGAG-3′).

The resulting PCR product was cleaved using NdeI and AccI and subcloned into the pAY430-SR-H_6_ plasmid cut likewise. To introduce the second Affibody molecule, the Z_HER2:342_ sequence was amplified a second time from pAY430-Z_HER2:342_-SR-H_6_. This time, PCR primers that introduced a 5′-BamHI-site, followed by three N-terminal glycines and a 3′-AccI site (5´- CCATCGGATCCGGCGGTGGCGTAGATAACAAATTCAACAAAGAAATGC-3′ and 5´- GTCTACGTCTACTTTCGGCGCCTGAG-3′), were used. This PCR product was cleaved with BamHI and AccI and subcloned into the plasmid cut likewise, resulting from the first step of the cloning procedure. The final plasmid, pAY430-G_3_-Z_HER2:342_-Cys-Z_HER2:342_-SR-H_6_, codes for a dimeric Z_HER2:342_-variant with the following amino acid sequence:

GGGVDNKFNKEMRNAYWEIALLPNLNNQQKRAFIRSLYDDPSQSANLLAEAKKLNDAQAPKEFGSGSGSCPGSGGGVDNKFNKEMRNAYWEIALLPNLNNQQKRAFIRSLYDDPSQSANLLAEAKKLNDAQAPKVDGSGSGSLPETGGHHHHHH The sequence of the final DNA construct was verified using sequencing (Microsynth, Göttingen, Germany).

Expression and subsequent IMAC purification of (Z_HER2_)_2:L_-Cys was performed following standard protocols for His_6_-tagged proteins [27]. After IMAC purification, the imidazole-containing elution buffer was changed into 10 mM NaOAc, pH 3.6 using PD-10 desalting columns (GE Healthcare, Uppsala, Sweden), and the protein was then lyophilized to a dry protein powder. Matrix-assisted laser desorption ionization mass spectroscopy (MALDI-MS) on a MALDI TOF/TOF analyzer (Sciex, Framingham, MA, USA) and electrospray ionization-mass spectrometry (ESI-MS) (Thermo Ultimate 3000, Thermo Fisher Scientific, Waltham, MA, USA, and Bruker Impact II, Bruker Daltonics, Billerica, MA, USA) were used to verify the molecular weight of the IMAC-purified (Z_HER2_)_2:L-_Cys. The sortase A-variant used to catalyze the cyclization reaction, P94S/D160N/K196T-sortase A, or sortase A^3^* in short, was expressed using a mutated variant of the pGBMCS-SortA vector provided by Addgene (Addgene plasmid no. 21931) [28], and purified as previously described by our group [29].

### 2.2. Template-Based Structure Prediction

A homology model of the linear dimer was generated by submitting the amino acid sequence to the fully automated SWISS-MODEL workspace on the ExPASy server. Available online: https://swissmodel.expasy.org (accessed on 26 May 2020) [30].

### 2.3. Reduction, Capping and Purification of (Z_HER2_)_2:L_

Lyophilized protein powder (10 mg) was dissolved in 1.5 mL of 200 mM ammonium bicarbonate, 3 M guanidine hydrochloride (GdnHCl); pH 8.5. The reducing agent, tris(2-carboxyethyl)phosphine (TCEP), was added to a final concentration of 50 mM from a frozen stock solution (500 mM TCEP in water, pH 7.0) to reduce any intermolecular disulfide bridges, and the sample was left to reduce on a thermo block set at 55 °C for 1 h. Following reduction, the protein denaturant GdnHCl was removed using a PD-10 column equilibrated with 200 mM ammonium bicarbonate, pH 8.5, and TCEP to a final concentration of 10 mM was immediately added to the eluted protein to maintain the cysteines in reduced condition. To cap the reduced cysteines, iodoacetamide (IAA) was added to a final concentration of 17 mM from a newly prepared stock solution (375 mM IAA in 200 mM ammonium bicarbonate, pH 8.5). After a 30 min reaction time, at RT and shielded from light, excess iodoacetamide was removed using PD-10 columns equilibrated in sortase A reaction buffer without Ca^2+^ (50 mM HEPES, 150 mM NaCl, pH 7.5). Half of the carbamidomethylated protein batch (~5 mg) was purified using reversed phase-high performance liquid chromatography (RP-HPLC) on a Zorbax C18 column (300SB-C18, 9.4 × 250 mm, 5 μm particle size; Agilent, Santa Clara, CA, USA) with an elution gradient rising from 25 to 55% B in 30 min (A: 0.1% trifluoroacetic acid (TFA) in milli-Q water; B: 0.1% TFA in acetonitrile) and a flow rate of 3 mL/min. The molecular weight of the iodoacetamide-reacted and linear (Z_HER2_)_2:L_ was verified using MALDI-MS and ESI-MS. HPLC-fractions containing the correct product were pooled and lyophilized.

### 2.4. Optimization of Sortase A-Mediated Head-to-Tail Cyclization

Reducing SDS-PAGE analysis was used to evaluate the optimal protein and enzyme concentrations for intramolecular head-to-tail cyclization. In an effort to minimize the formation of higher molecular weight species, (Z_HER2_)_2:L_ was diluted to a concentration range between 68 and 1.7 μM, while the sortase A^3^* concentration was kept at 5 μM. After a 30 min reaction at 37 °C, samples were added to a 15-well NuPAGE 4–12% Bis-Tris gel (Life Technologies, Carlsbad, CA, USA). The gel was run under reducing conditions and stained using GelCode^TM^ Blue Safe Protein Stain (Pierce, Rockford, IL, USA) according to the manufacturer’s instructions. The optimal (Z_HER2_)_2:L_ concentration was chosen as the highest protein concentration that did not produce higher molecular weight species visible on a Coomassie-stained SDS-PAGE gel.

In the next step, the concentration of (Z_HER2_)_2:L_ was maintained constant at 1.7 μM, while the concentration of sortase A^3^* was altered between 0.08 and 5 μM. The samples were left at 37 °C for 20–24 h. Approximately 2.5 μg (Z_HER2_)_2:L_ from each sample was applied to an SDS-PAGE gel, and the gel was run, stained, and inspected as described for the previous gel.

### 2.5. Cyclization and Purification of (Z_HER2_)_2:C_

In a typical cyclization reaction, 300 nmol (~5 mg) of alkylated (Z_HER2_)_2:L_ was diluted to 1.7 μM in 176 mL sortase A reaction buffer containing 3 μmol NiCl_2_. The cyclization reaction was initiated by the addition of sortase A^3^* to a final concentration of 0.63 μM, and the reaction was left to proceed at 37 °C for 17–20 h. Following cyclization, the protein sample was concentrated, and buffer was exchanged using Amicon Ultra-15 filtration units with a 3 kDa cut-off (Merck Millipore, Darmstadt, Germany) down to ~10 mL in 50 mM HEPES, 150 mM NaCl; pH 7.5. The cyclic protein was purified using a “reversed IMAC step” on a HisPur Cobalt resin (Pierce, Rockford, IL, USA) equilibrated with 50 mM HEPES, 150 mM NaCl; pH 7.5. Unreacted linear protein and His-tagged sortase A^3^* binds to the column matrix, while cyclized protein, lacking the C-terminal His_6_-tag, is eluted in the flow through. Fractions absorbing at 280 nm were pooled and buffer was exchanged to 10 mM NaOAc, pH 3.6 using PD-10 columns before protein lyophilization. The cyclic protein was purified using RP-HPLC, using the same conditions as previously described for the linear (Z_HER2_)_2:L_ protein but with a slightly different gradient (35–45% B over 20 min). For representative chromatograms of the RP-HPLC purification, see Figure S1. The molecular weight of the purified cyclic iodoacetamide-capped Z_HER2_-dimer, hereafter called (Z_HER2_)_2:C_, was verified using MALDI-MS, and HPLC fractions containing the correct product were pooled and lyophilized.

SDS-PAGE analysis was used to determine the efficiency of the sortase A-mediated cyclization reaction, and the apparent molecular weight of the linear and cyclic IAA-capped Z_HER2_-dimers. The samples were mixed with 5.2 µL glycerol and 4 µL 5× loading buffer containing β-mercaptoethanol, and 1× sortase reaction buffer was added to reach a volume of 20 µL. PageRuler^TM^ Plus (Thermo Fischer Scientific, Vilnius, Lithuania) pre-stained protein ladder was used as molecular weight standard. The samples contained approximately 2.5 µg of protein and were heated for 5 min at 95 °C and loaded on to a 10-well NuPAGE 4–12% BT 1.0 gel (Life Technologies, Carlsbad, CA, USA). The gel was run in an Novex Mini-Cell (Life Technologies, Carlsbad, CA, USA). for 35 min at 180 V in 1× MES buffer. The gel was stained with GelCode^TM^ Blue Safe Protein Stain (Pierce, Rockford, IL, USA) and scanned. Gels used for protein band intensity measurements were color-adjusted using the Black & White adjustment tool in Adobe Photoshop 2021 (Adobe), and protein band pixel intensities were analyzed using the open-source software ImageJ, version 1.8.0_172 Available online: http://imagej.nih.gov (accessed at 10 May 2021). The relative mobility (Rf; migration distance of the protein/migration distance of the dye front) of the proteins in the molecular weight standard was plotted against the logarithm of their molecular weights, and a linear equation was fitted to the data points using Excel for Mac, version 16.48 (Microsoft, Redmond, WA, USA). The linear calibration curve was used to determine the apparent molecular weights for the IAA-capped linear and cyclic Z_HER2_-dimers.

### 2.6. Production and Purification of (Z_HER2_)_2:C_-DL594

A mass of 5 mg of (Z_HER2_)_2:L_-Cys was cyclized and purified using the optimized protocol outlined for (Z_HER2_)_2:L_ in Section 2.5. above, with minor changes described here. To keep the free cysteine in (Z_HER2_)_2:L_-Cys in a reduced state, 10 mM of TCEP was added to the sortase reaction buffer during the 22 h cyclization reaction. Following cyclization, the protein was concentrated on Amicon Ultra-15 filtration units with 10 kDa MWCO (Merck), and after a “reversed IMAC” treatment, as described in Section 2.5., the cyclic Z_HER2_ dimer, (Z_HER2_)_2:C_-Cys, was buffer-exchanged to 10 mM NaOAc, pH 3.6 using PD-10 columns and lyophilized to a dry protein powder. 

For maleimide labeling, 1 mg (~60 nmol) of lyophilized protein was dissolved in 0.5 mL of 200 mM ammonium bicarbonate, 3 M GdnHCl; pH 8.5 with 50 mM TCEP, and the sample was left to reduce on a thermo block set at 55 °C for 1 h. The reduction buffer was removed using a NAP-5 column (GE healthcare, Uppsala, Sweden), and the protein was eluted in 1 mL of 20 mM Tris buffer, pH 7.0, containing 10 mM TCEP. One milligram of DyLight 594 maleimide fluorescent dye (Pierce, Rockford, IL, USA) was dissolved in 100 μL of *N*,*N*-dimethylformamide (DMF) and added to the reduced protein at 18 molar excess relative to the sulfhydryl group in the protein. Following a 17 h reaction at room temperature, the sample was dialyzed in a dialysis tube with a 6–8 kDa MWCO (Spectra/Por Membrane; Spectrum Laboratories Inc., Rancho Dominguez, CA, USA), against 4 L of 10 mM potassium phosphate buffer with 15 mM KCl, pH 7.2 at 4 °C for 24 h. A final RP-HPLC step using the same HPLC setup described in Section 2.3. but using a slightly different gradient (30–45% B over 15 min) was added to remove remaining unreacted fluorescent dye and sortase A from the cyclic DyLight 594-labeled Z_HER2_-dimer. For a representative chromatogram of the RP-HPLC purification, see Figure S2. The molecular weight of (Z_HER2_)_2:C_-DL594 was verified using MALDI-MS, and HPLC-fractions containing the correct product were combined and lyophilized. The protein concentration and the degree of labeling were estimated by measuring the absorbance at 280 nm and 593 nm, following the manufacturer’s protocol.

### 2.7. Circular Dichroism Spectroscopy

Circular dichroism (CD) studies of the linear and cyclic proteins were performed using a Chirascan CD spectrometer (Applied Photophysics, Leatherhead, UK) equipped with a Peltier temperature-controlled cuvette holder and a thermal sensor that records the temperature in the sample cuvette. Both proteins were dissolved in 20 mM potassium phosphate buffer with 100 mM KCl (pH 7.4) at a concentration of about 0.2 mg/mL, and all CD measurements were performed in a capped quartz cuvette with a 0.1 cm path length. Far-UV spectra of the proteins were recorded by scanning the ellipticity in the 195–260 range, and were converted to molar ellipticity before data processing. The mean residue ellipticity (MRE) is given by:

(1) $$\mathrm{MRE}=\frac{\theta_{\mathrm{obs}}}{10\times l\times c\times n}$$

where θ_obs_ is the measured ellipticity in degrees, l is the cuvette path length in centimeters, c is the protein concentration in molar quantities, and n is the number of amino acids in each protein. The fraction helix F_H_ was calculated from the mean residue ellipticity at 222 nm, MRE_222_, by the method of Scholtz et al. [31]:

(2) $$F_{H}=\frac{{\mathrm{MRE}}_{222}-{\left[\theta \right]}_{C}}{{\left[\theta \right]}_{H}-{\left[\theta \right]}_{C}}$$

Complete helix [θ]_H_ and complete random coil [θ]_C_ are expressed in deg cm^2^ dmol^−1,^ and are given by:

(3) $${\left[\theta \right]}_{H}=-40,000\times \left(1-\frac{2.5}{n}\right)+100\times T$$

(4) $${\left[\theta \right]}_{C}=640-45\times T$$

T is the temperature expressed in °C, and n is the number of residues in the protein [31]. Thermal unfolding was monitored by recording the ellipticity at 222 nm, θ_222_, in millidegrees as a function of temperature. The temperature was increased between 20 and 90 °C in 0.1 °C increments at a speed of 5 °C/min. 

Fraction folded (F_N_) as a function of temperature was calculated using the following equation, assuming a two-state unfolding behavior:

(5) $$F_{N}=\frac{\theta_{222}-\theta_{N}}{\theta_{N}-\theta_{D}}$$

where θ_N_ and θ_D_ are the ellipticity at 222 nm, in millidegrees, of the native and the denatured state, respectively. 

After denaturation, the temperature in the cuvette was returned to 20 °C and the far-UV spectra of the proteins were rescanned to ensure folding reversibility.

### 2.8. Surface Plasmon Resonance (SPR)-Based Binding Analysis

The binding kinetics of the interactions between the cyclic and linear proteins and HER2 were analyzed on a Biacore T200 system (GE Healthcare, Uppsala, Sweden). A carboxymethylated, dextran-coated CM5 chip was activated with EDC/NHS and HER2-Fc (Her2/ERBB2 Protein, Human, Recombinant (hFc Tag); Sino Biological Inc., Beijing, China) was immobilized on the surface with an immobilization level of 760 RU. The analytes were diluted in phosphate-buffered saline with 0.5% Tween-20 (PBS-T), pH 7.4, to 5 different concentrations, 500, 167, 56.6, 18.5 and 6.2 nM, and were allowed to flow over the surfaces at 50 µL/min in a single-cycle setup with a 5 min association phase and a final 120 min dissociation time for 2 replicates. The equilibrium dissociation constant (K_d_) was determined from a 1-to-1 binding model.

### 2.9. Protease Digestion Assay

The difference in stability towards protease degradation of the cyclic and linear proteins was demonstrated with a mixture of the endopeptidases α-chymotrypsin (≥40 U mg^−1^) and trypsin (12,443 U mg^−1^), called pancreatin, and the exopeptidase carboxypeptidase A (70 U mg^−1^). All proteins were extracted from bovine pancreas and bought from Sigma-Aldrich (Saint Louis, MO, USA). The sample proteins were diluted in 0.1 M Tris-Cl (pH 8.5) to a concentration of 50 µM. The cleavage was measured after 0, 5, 15, 30 and 60 min. In the pancreatin assay, 54 µL protein was mixed with 102 µL of pancreatin in PBS (pH 7.4) to a final concentration of 33 µM protein, 0.4 µg/mL α-chymotrypsin and 0.9 µg/mL trypsin. The digestions were performed at 37 °C and the reactions were stopped by the addition of 9 µL 10% TFA. The samples were analyzed by RP-HPLC (1200 series, Agilent Technologies, San Diego, CA, USA) at 35 °C on a Zorbax C18 analytical column (300SB-C18, 4.5 × 15 mm, 3.5 µM particle size; Agilent, Santa Clara, CA, USA) with a flow rate of 1 mL/min over 20 min with a gradient of 0–80% B (A: 0.1% TFA in milli-Q water; B: 0.1% TFA in acetonitrile). The elution peaks were integrated and compared to the undigested sample at t = 0. The digestions were performed as technical triplicates, except for the 0 time point and the time points where 100% of the protein had been digested, where only one data point was gathered.

In the carboxypeptidase A assay, 10 µL of protein sample in 0.1 M Tris-Cl (pH 8.5) was mixed with 10 µL of carboxypeptidase A in 0.1 M Tris-Cl (pH 8.5) to a final concentration of 25 µM protein and 0.5 µM carboxypeptidase A. The reactions were stopped with the addition of 1 µL of 10% TFA, and the samples were analyzed by MALDI-MS (MALDI TOF/TOF analyzer, Sciex) to determine the amount of undigested protein.

### 2.10. Cell Culture and Treatment

A human ovarian cell line (SKOV-3) that presents a high expression of HER2 surface receptors and a human breast cancer cell line (MCF-7) with a low expression of HER2 receptor were cultured at 37 °C in a 5% CO_2_ humidified atmosphere in Dulbecco’s modified Eagle medium (DMEM) (Gibco, Thermo Fisher Scientific, Uppsala, Sweden) supplemented with 10% fetal bovine serum, 0.1 mM non-essential amino acids (Gibco, Thermo Fisher Scientific, Sweden), 2 mM L-glutamine (Gibco, Thermo Fisher Scientific, Sweden) and 1% penicillin/streptomycin (Gibco, Thermo Fisher Scientific, Sweden). Coverslips were added in a 12-well plate and treated with poly-L-lysine (Sigma-Aldrich, Stockholm, Sweden) (0.1 mg/mL) for 15 min at room temperature, and 100,000 SKOV-3 cells were seeded on the coverslips. After an overnight incubation, the media was removed and replaced with fresh DMEM containing (Z_HER2_)_2:C_ (1.2 µM), and the cells were incubated for 5 min at 37 °C. (Z_HER2_)_2:C_-DL594 was then added to the cells (2.4 nM) which were then incubated at 37 °C for 1 h. A positive control was also prepared with cells incubated with only (Z_HER2_)_2:C_-DL594 (2.4 nM) for 1 h at 37 °C. The medium was then removed, and after 3 washes with PBS, a solution of 4% paraformaldehyde (PFA) in PBS (Alfa Aesar, Thermo Fisher Scientific, Sweden) was added to each well (15 min, room temperature) to fix the cells. The PFA solution was removed, and the cells were washed once with PBS. All the cells were then treated with 4′,6-diamidino-2-phenylindole (DAPI) (Roche, Merck, Sweden) (1 µg/mL, 5 min, room temperature) for nuclear staining. After a wash with PBS, the coverslips were then collected and mounted on a microscopy slide before sealing them with nail polish.

On an 8-well microscopy slide (Millicell EZ, Merck, Sweden), 20,000 SKOV-3 cells were seeded and allowed to grow overnight. On the day of treatment, the media were removed and replaced with fresh DMEM containing trastuzumab (Herceptin^®^, Roche, Sweden) (1 mg/mL) and incubated for 5 min at 37 °C before the addition of (Z_HER2_)_2:C_-DL594 (2.4 nM) and another hour of incubation at 37 °C. The cells were then washed, fixed, and mounted in the same fashion as previously. On another 8-well microscopy slide, 20,000 MCF-7 cells were seeded and allowed to grow for 2 days. The medium was then replaced with fresh DMEM containing (Z_HER2_)_2:C_-DL594 (2.4 nM) and the cells were incubated for 1 h at 37 °C. The cells were then again washed, fixed, and mounted in the same fashion as the SKOV-3 cells.

All cells were visualized using a Nikon Plan Fluor 10× Ph1 DL optic (BergmanLabora AB, Danderyd, Sweden) and the images were captured with an Andor Zyla VSC-05780 camera (BergmanLabora AB, Danderyd, Sweden) coupled to the software NIS-Element. The images were then processed with ImageJ, version 1.8.0_172.

## 3. Result and Discussion

### 3.1. Design and Production of Linear and Cyclic Anti-HER2 Dimers

**Figure 1:**
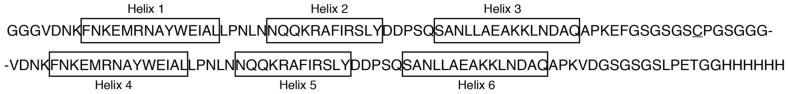
Amino acid sequence of the dimeric HER2-binding Affibody (Z_HER2_)_2:L_-Cys. The protein construct is designed to be made up of two domains with three-helix folds connected by a 15-residue linker containing a unique cysteine, underlined in the sequence, for simple labeling of the construct with thiol-reactive compounds. The dimer has three N-terminal glycines and carries a C-terminal sortase A recognition motif (LPETG) to make the construct a substrate for sortase A-mediated cyclization. The hexahistidine tag in the linear construct is used for IMAC purification of the *Escherichia coli*-expressed construct, but is cleaved off during the cyclization reaction. Helical regions in a crystal structure of the parental monomeric Z_HER2:342_ Affibody are indicated by helices 1–3 and 4–6, respectively [32].

A Z_HER2:342_-dimer with three N-terminal glycines and a C-terminal sortase A recognition sequence, LPETGG, followed by a His_6_-tag for IMAC purification was successfully expressed and purified from *E. coli* (Figure 1).

**Figure 2:**
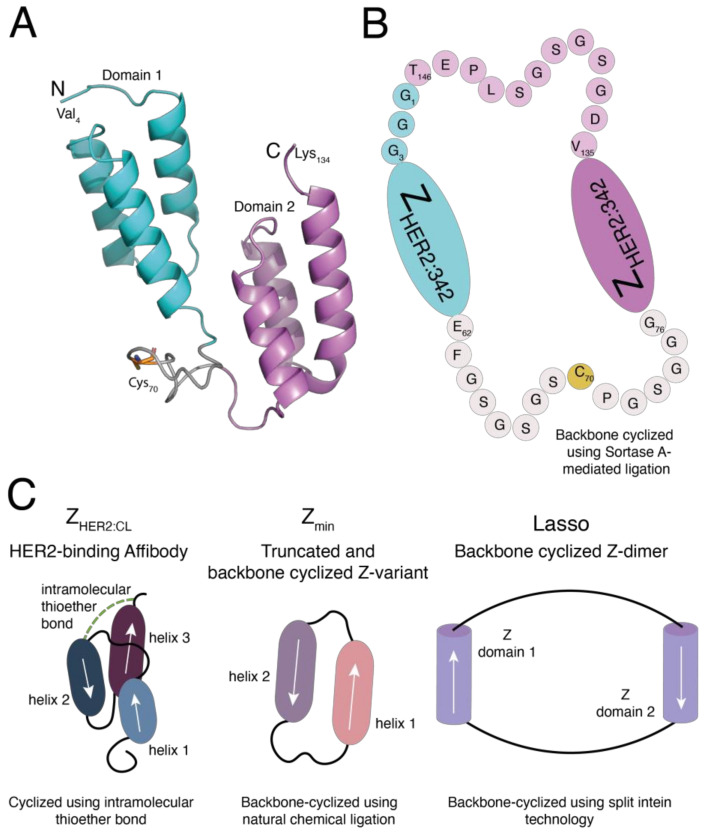
(**A**) Homology model of (Z_HER2_)_2:L_-Cys generated with the SWISS-MODEL protein modelling server [30] based on a crystal structure of two tandem B-domains connected by a conserved linker (PDB code: 4NPF [33]). Residues 1–3, i.e., the three N-terminal glycines, and C-terminal residues 135–154, containing the sortase A recognition site and the His6-tag, were not included in this homology model. The unique cysteine, C_70_, is predicted to be situated in the linker region between domains 1 and 2. (**B**) Schematic structure of the cyclic Z_HER2_-dimer, (Z_HER2_)_2:C_. Sortase A-mediated cyclization of the dimeric protein results in the formation of a native peptide bond between Gly_1_ and Thr_146_. (**C**) Schematic illustrations of three different approaches to intramolecular crosslinking or backbone cyclization of Affibody molecules previously published by our group and others. The HER2-binding Z_HER2:CL_ was synthesized using solid phase peptide synthesis (SPPS) and has an intramolecular thioether bond going from a cysteine residue in the loop between helices 1 and 2 and the chloroacetyl-modified side chain of the C-terminal lysine residue [24]. Z_min_ is a truncated version of the Z-domain, in which the two IgG-binding helices 1 and 2 are joined by a peptide bond. Z_min_ was prepared using SPPS and backbone-cyclized using natural chemical ligation [25]. Lasso is a recombinantly expressed Z-domain dimer. The two IgG-binding Z-domains are joined by flexible linkers and the construct is backbone-cyclized using split-intein technology [26]. The schematic drawings of Z_HER2:CL_, Z_min_ and lasso are based on information found in references [24,25,26].

A SWISS-MODEL homology model of the linear Z_HER2_-dimer, (Z_HER2_)_2:L_-Cys, generated using a crystal structure of two identical B domains connected by a short and conserved linker as a template (PDB code: 4NPF [33]) is shown in Figure 2A. The homology model predicts that domain 1 and domain 2 fold independently into two three-helical bundle domains connected by a flexible linker. A unique cysteine residue is located in the middle of this flexible linker and is intended to work as a chemical handle for potential modifications of the protein with, for example, fluorophores or chelators for radiometal labeling. For studies of the nonlabeled protein, we chose to derivatize the cysteine with iodoacetamide (IAA) to block potential intermolecular protein–protein disulfide bond formation. This IAA-capped variant of the protein, (Z_HER2_)_2:L_, is referred to as the linear dimer throughout the text. Upon treatment with sortase A, a native peptide bond is formed between Thr_146_ and Gly_1_, and the cyclic dimer, (Z_HER2_)_2:C_, is formed (Figure 2B). For reference, the designs of three cyclic Affibody variants described earlier in the literature [24,25,26] are schematically shown (Figure 2C). It can be noted that the two previously described cyclic HER2-binding Affibody molecules are monomeric or truncated versions of the protein, not taking advantage of the possible avidity effects that are expected from dimeric constructs. In contrast, the bivalent “lasso” construct formed by cyclization of two Z domains is designed for simultaneous binding of the two domains to IgG. The new design presented here combines the use of dimeric Affibody constructs with a backbone cyclization strategy, applied to the therapeutically relevant HER2-binding Affibody molecule.

To drive the sortase A-catalyzed reaction towards head-to-tail intramolecular cyclization, and to avoid the formation of multimers and cyclic dimer-of-dimers, the linear dimer was diluted to 1.7 μM during the cyclization reaction. Multimer formation and cyclization of higher molecular weight species during sortase A mediated ligation of proteins carrying both N-terminal glycines and a C-terminal LPETG recognition sequence has previously been observed by several groups [19,22]. In our initial cyclization trials, a relatively high (5 μM) concentration of sortase A^3^* was used to convert the linear dimer to (Z_HER2_)_2:C_. This enzyme concentration has previously been used for intermolecular GGG-PNA and GGG-DNA conjugation to Affibody molecules with a C-terminal LPETGG-H_6_-tag [27,34]. Following optimization of the circularization reaction, the sortase A^3^* concentration was reduced to 0.63 μM, while the reaction time was prolonged to 20–24 h at 37 °C.

**Figure 3:**
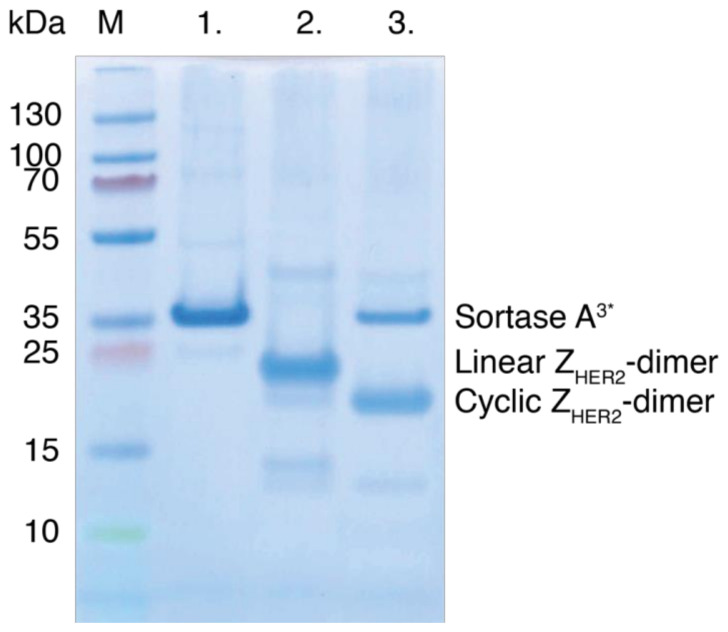
SDS-PAGE analysis of sortase A-mediated cyclization of the Z_HER2_-dimer. M: Molecular weight marker. Lane 1: Sortase A^3^*, lane 2: (Z_HER2_)_2:L_, and lane 3: unpurified sample taken after a 20 h sortase A-mediated cyclization reaction.

Using these optimized reaction conditions, the efficiency of the cyclization reaction was estimated to be >95% as judged by SDS-PAGE (Figure S3), and no higher molecular weight species, indicative of sortase A-mediated multimerization, are visible after Coomassie staining (Figure 3). Both the linear and the cyclic Z_HER2_-dimers run as proteins with higher apparent molecular weights on SDS-PAGE (~25 and ~21 kDa, respectively) than expected by their amino acid sequence. The noticeably faster migration rate of the cyclic protein compared to the linear dimer cannot be explained solely by the loss of eight amino acid residues in the cyclization reaction, which suggests that the cyclic protein has a more compact hydrodynamic shape. Similar changes in hydrodynamic radii have previously been seen for most, but not all, head-to-tail and side-chain cyclized proteins characterized by SDS-PAGE thus far [16]. The cyclic Z_HER2_-dimer was purified with an IMAC-purification step following cyclization, and the purity of both the linear and the cyclic proteins were polished with a final RP-HPLC step (Figure S1). The identities of the purified proteins were confirmed using ESI-MS (Table 1, Figures S4–S6) and MALDI-TOF (Table 1, Figures S7 and S8).

### 3.2. Circular Dichroism

**Figure 4:**
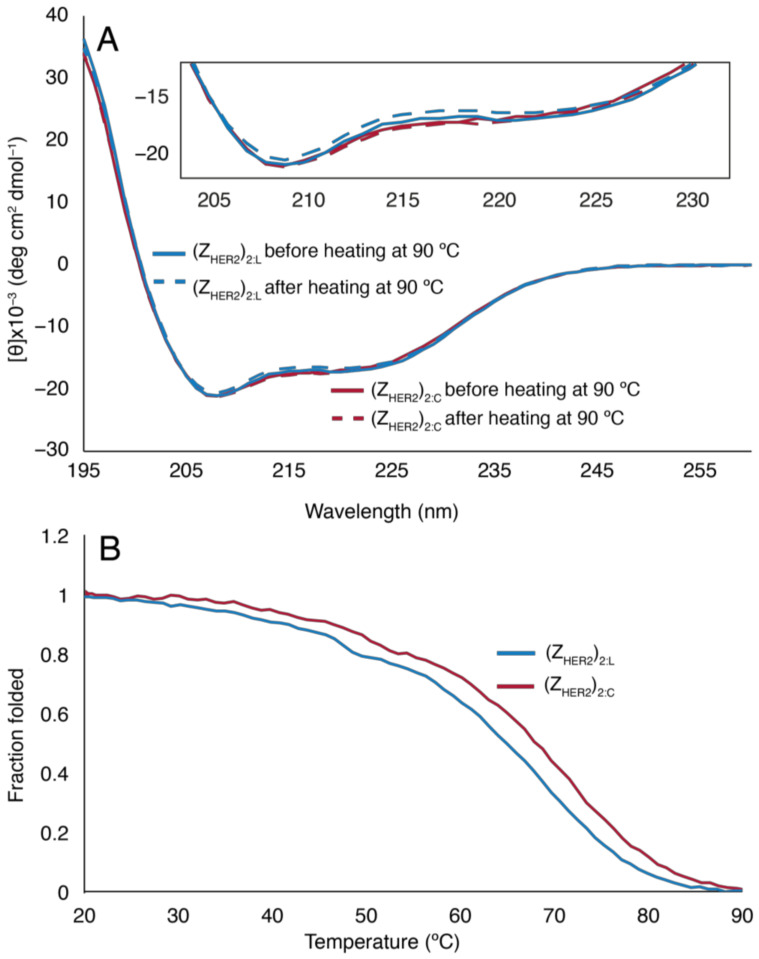
(**A**) Circular dichroism (CD) expressed as molar ellipticity of linear (Z_HER2_)_2:L_ (blue) and (Z_HER2_)_2:C_ (red) before (solid line) and after (dashed line) refolding after thermal melt at 90 °C. Inserted is a close-up of the CD spectra in the 205–230 nm region. (**B**) Fraction folded protein as a function of temperature for (Z_HER2_)_2:L_ (blue) and (Z_HER2_)_2:C_ (red). The melting temperature, T_m_, of the linear dimer was 65 °C and it was elevated to 68 °C in the cyclic dimer.

The molar ellipticity [θ] for the linear and cyclic dimeric proteins looked very similar in the far-UV range, both before and after thermal melt at 90 °C (Figure 4A). MRE_222_, the mean residue ellipticity at 222 nm, was −17,100 and −17,900 deg cm^2^ dmol^−1^ for the linear and the cyclic Z_HER2_-dimer, respectively. Assuming that only amino acids in helical conformation contribute to the CD signal at 222 nm, this corresponds to about 45 and 48% helical residues in the linear and cyclic dimer, respectively [31,35], or, ~70 residues in each dimeric protein having a helical backbone conformation. An analogous calculation from the CD spectra of the parental 58-residue Z_HER2:342_ monomer suggested a helical content of 73% or ~42 residues, and free solution NMR structures of the monomer showed 39–41 amino acids in helical conformation depending on the conformational state of helix 1 [32]. Compared to the parental protein, our Z_HER2:342_-dimer constructs had fewer helical residues per monomer but the head-to-tail cyclization did not appear to have an impact on the number of residues adopting a helical configuration according to CD data at 222 nm. Upon cyclization, the thermal melting temperature, T_m_, of the dimer was increased by 3 °C from 65 °C to 68 °C (Figure 4B). This modest increase in ΔT_m_ is in agreement with published values for other sortase A-catalyzed backbone cyclized proteins, i.e., interferon α2, granulocyte colony-stimulating factor and human growth factor, which all increased their melting temperature by 2.1–4.9 °C relative to their linear counterparts [21,22].

### 3.3. Surface Plasmon Resonance (SPR)-Based Biosensor Analysis

**Figure 5:**
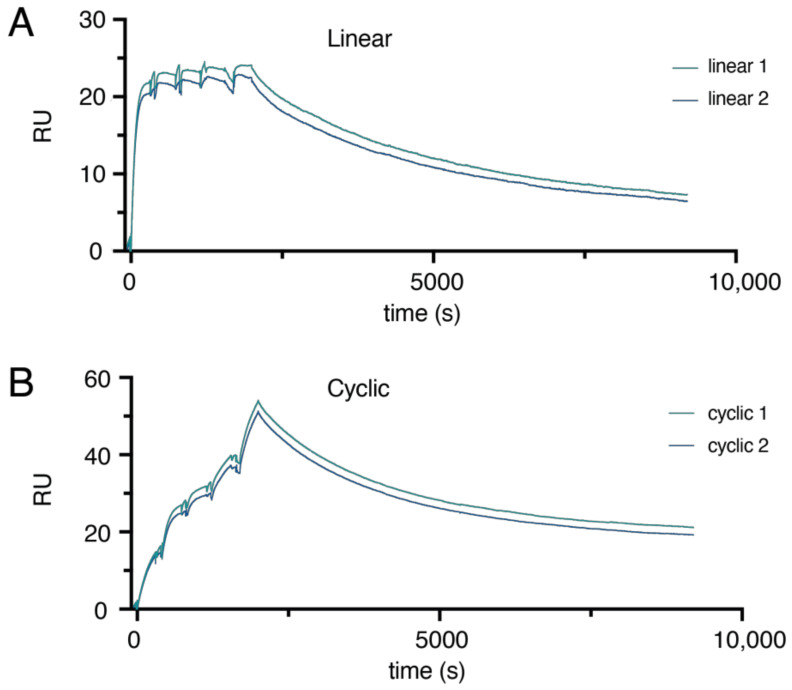
Evaluation of kinetic titration series of (**A**) the linear dimer (Z_HER2_)_2:L_ and (**B**) the cyclic dimer (Z_HER2_)_2:C_ binding to immobilized HER2-Fc.

The binding between the cyclic and linear protein to HER2 was analyzed using surface plasmon resonance (SPR) and the kinetic titration method (Figure 5). HER2-Fc was immobilized on the surface of a CM5 chip, and the cyclic and linear proteins were allowed to flow over the chip at different concentrations. Kinetic parameters were estimated from fitting the sensorgrams using a 1:1 binding model even though the Z_HER2_-proteins are dimeric because a 1:1 binding model rendered a good fit and reasonable values for the kinetic parameters. The apparent dissociation constant, K_d_, for the linear dimer, (Z_HER2_)_2:L_, binding to the HER2 receptor was estimated to be 5 pM, which is comparable to a previously determined value of 22 pM for the monomeric Z_HER2:342_-protein [2]. The cyclic protein, (Z_HER2_)_2:C_, was estimated to have an apparent affinity of 750 pM for the receptor, which is significantly weaker than the linear dimer, but the protein still retained a tight sub-nanomolar binding to HER2-Fc. This drop in apparent affinity was somewhat unexpected, because the CD results suggest that the Affibody domains have a similar helical folded structure in both constructs. It has previously been shown that N-terminal modifications of the HER2-binding Affibody molecule can affect its binding affinity. When extending the N-terminus of a synthetic HER2-binding Affibody molecule with the peptidic mercaptoacetyltriglycyl (MAG3) radionuclide chelator, the K_d_ dropped from 80 pM to 200 pM [36]. In another study, a recombinant dimeric HER2-binding Affibody molecule was fused N-terminally or C-terminally to an albumin-binding domain, and although no kinetic parameters were reported, it was shown that the N-terminal fusion had a significant, negative impact on the binding to HER2 [37]. A possible explanation for the observed differences in affinity could thus be that the amino acids flanking the Affibody domains in the cyclic and linear constructs are partly different.

Interestingly, our SPR data suggest that a slower on-rate is responsible for the weaker overall affinity of the cyclic dimer (k_a_: ~8.2 × 10^6^ and ~2.1 × 10^5^ M^−1^s^−1^ for the linear and the cyclic dimer, respectively).

The dissociation rate constant, k_d_, was more similar for the two proteins (k_d_: ~4.2 × 10^−4^ and ~1.6 × 10^−4^ s^−1^ for the linear and the cyclic dimer, respectively). The k_d_ values measured here for the dimers are close to the k_d_ value (2.9 × 10^−4^ s^−1^) previously published for the dissociation of the monomeric Z_HER2:342-_SR-H_6_ protein from HER2-Fc [27]. Thus, no clear avidity effects are seen for the dissociation of the dimeric protein variants from HER2-Fc receptor compared to a monomeric variant of the same Affibody molecule. A prerequisite for bivalent binding is that the two binding domains simultaneously bind to the receptor, but because binding of the first domain can be sterically hindered by the presence of the second domain, reduced affinity can in fact be seen in certain dimeric constructs. In a recent study aiming at the development of radiolabeled CD20-targeting single-domain antibody fragments (sdAbs), it was shown that the apparent binding affinity decreased when two such binding domains were fused [38].

For HER2-binding Affibody molecules, dimeric constructs have been used to increase the apparent affinity for in vivo applications [39]. Altai et al. compared a monomeric and a dimeric variant of an anti-HER2 Affibody molecule Z_HER2:2891_ conjugated to the toxin DM1, and saw clear avidity effects on the off-rate in the SPR sensorgram for the dimeric variant binding to immobilized HER2 receptor [40]. Their dimeric construct contained a short five residue (-GGGGS-) linker between the two HER2-binding Affibody domains, suggesting that the 15-residue linker between helix 3 and helix 4 (Figure 1) used in this study could be too long and/or too flexible to give rise to an observable avidity effect when binding to the HER2 receptor.

For Affibody molecules binding to other targets than HER2, longer linkers may instead be beneficial. Zhou and coworkers used split-intein technology to produce a dimeric and backbone-cyclized Z-domain capable of binding to human IgG1 with increased affinity compared to the original monomeric protein [24], suggesting that the two domains can bind simultaneously on opposite sides of the symmetrical IgG1 dimer. A linear precursor protein, with a 17-residue linker separating the two Z-domains, showed a 10–12-fold higher affinity for human IgG1 compared to monomeric Z, and importantly, the subsequent backbone cyclization reaction, in which a significantly longer second 45–54 residue linker was formed, did not have a detrimental effect on the antibody binding affinity according to SPR and flow cytometry data [26].

Taken together, the data from this study and the literature suggest that the design of cyclic dimers is complex and that sterical hindrance, conformational strain, and suboptimal positioning of the domains relative to the binding target will affect the apparent affinity. It is possible that the binding affinity of our cyclic Z_HER2:342_ dimer could be improved by optimizing the lengths of the two connecting linkers independently. The apparent sub-nanomolar affinity for HER2 achieved for the construct is still likely sufficient for many applications.

### 3.4. In Vitro Digestion Assay

**Figure 6:**
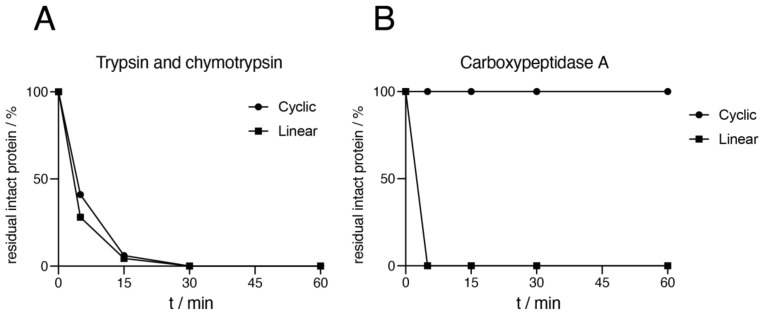
In vitro digestion of (Z_HER2_)_2:L_ and (Z_HER2_)_2:C_. (**A**) Digestion with trypsin (0.9 µg/mL) plus chymotrypsin (0.4 µg/mL). (**B**) Digestion with carboxypeptidase A (0.5 µM).

The relative proteolytic stability towards endopeptidase and exopeptidase degradation was determined through two separate digestion assays. A mixture of trypsin and chymotrypsin was used for the endopeptidase assay, and carboxypeptidase A was used for the exopeptidase assay. The digestion of the proteins was measured over time and was followed by RP-HPLC for the trypsin and chymotrypsin digestion assay, where the areas of the peaks corresponding to intact protein were integrated and plotted. The carboxypeptidase A digestion assay did not yield a digestion product that was possible to separate from the undigested peak, and the digestion was measured by MALDI-MS (Figure 6). There was no apparent difference in proteolytic stability between the cyclic and linear dimers in the endopeptidase assay, where both samples were totally degraded after 30 min (Figure 6A). In the exopeptidase assay, the linear dimer was completely degraded after 5 min, while the cyclic dimer showed no degradation after 60 min (Figure 6B). This demonstrated the success of cyclization as an effective strategy to protect a protein against exopeptidase degradation, which is dependent on free protein termini. There was no improvement in endopeptidase stability of the cyclic protein when compared to the linear version. Globular proteins with compact and well-folded hydrophobic cores are generally more resistant towards proteolytic attacks than those with more flexible loop regions [8], and the reduction in hydrodynamic radii observed here upon cyclization was apparently not sufficient to lead to a noticeable effect on the endopeptidase stability.

### 3.5. Fluorescence Microscopy

**Figure 7:**
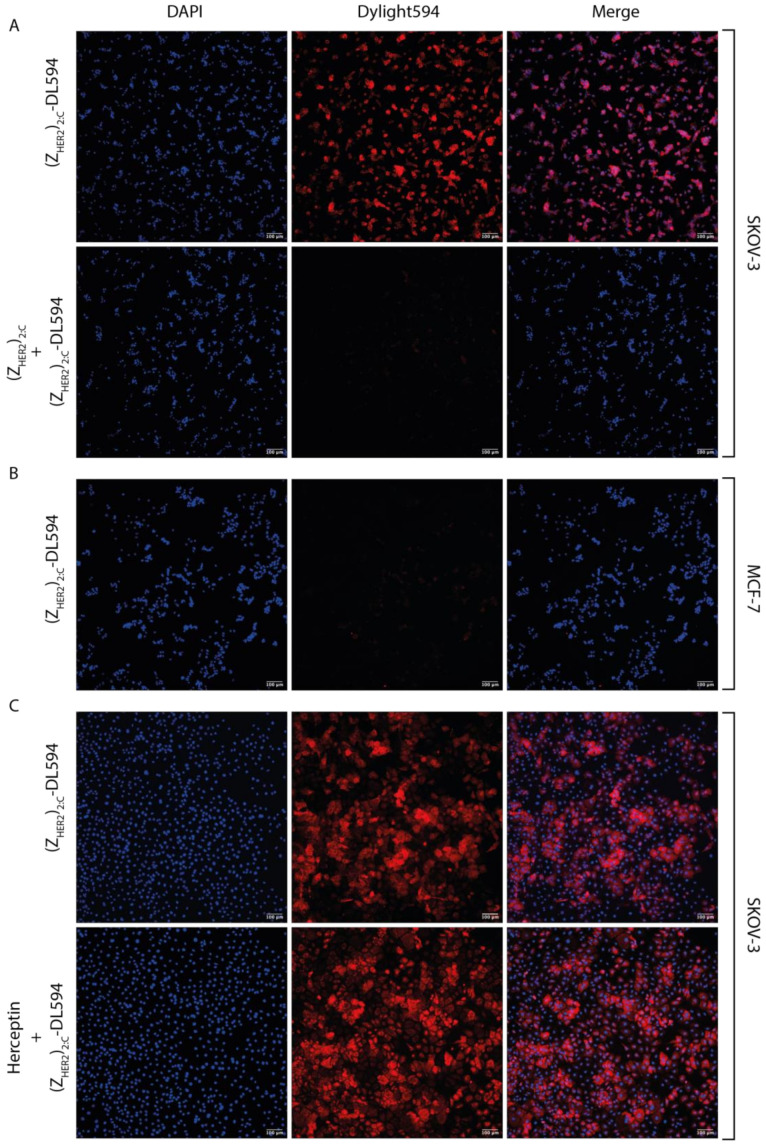
Fluorescent microscopy images of either SKOV-3 cells or MCF-7 cells with DAPI-stained nuclei (blue) treated with 2.4 nM of (Z_HER2_)_2:C_-DL594 (red). (**A**) SKOV-3 cells treated with (Z_HER2_)_2:C_-DL594 as a positive control in the first row. In the second row, SKOV-3 cells pre-incubated with unlabeled (Z_HER2_)_2:C_ (1.2 µM) before addition of (Z_HER2_)_2:C_-DL594. (**B**) MCF-7 cells treated with (Z_HER2_)_2:C_-DL594. (**C**) SKOV-3 cells treated with (Z_HER2_)_2:C_-DL594 as a positive control in the first row. The second row presents SKOV-3 cells pre-incubated with trastuzumab (Herceptin; 1 mg/mL) before the addition of (Z_HER2_)_2:C_-DL594.

In order to evaluate the binding specificity for the HER2 surface receptor of the cyclic Z_HER2:342_ dimer, the construct was fluorescently labeled and added to HER2-rich SKOV-3 cells that were pre-treated with a saturating concentration of unlabeled cyclic Z_HER2:342_ dimer. The cells were then imaged using fluorescent microscopy. The addition of the unlabeled construct almost entirely abolished the binding of the fluorescently labeled cyclic dimer (Figure 7A), showing that the unspecific binding for the cyclic Z_HER2:342_ dimer was very low. This blocking proved the high specificity of our new construct for the HER2 receptor. More proof of the high specificity of the cyclic dimer is given in Figure 7B. MCF-7 cells present a low basal level of HER2 expression [41] and, upon treatment with the labeled cyclic Z_HER2:342_ dimer in the same conditions as the SKOV-3 cells, did not show any significant binding to the construct. The insignificant binding on MCF-7 cells indicates that in the absence of HER2 surface receptor, the cyclic dimer does not have any binding sites. A final experiment was prepared to ensure that the recognition site where the Affibody Z_HER2:342_ binds to HER2 receptor was not shifted by the cyclisation and the addition of the linker between the two monomers. SKOV-3 cells were pre-treated with a saturating concentration of the therapeutic monoclonal antibody trastuzumab (Herceptin^®^). It is known from previous studies that the Affibody Z_HER2:342_ and trastuzumab bind to non-overlapping sites in the HER2 receptor [32]. In Figure 7C, it can clearly be seen that the addition of a saturating concentration of trastuzumab does not affect the binding of the cyclic Z_HER2:342_ dimer. The trastuzumab blocking experiment thus suggests that the binding site in the HER2 receptor is retained for the cyclic Z_HER2:342_ dimer.

## 4. Conclusions

An enzyme-mediated strategy has been investigated for the generation of compact, conformationally constrained, head-to-tail cyclic Affibody dimers lacking free N- and C-termini. The protocol is based on using sortase A, which is a transpeptidase originally isolated from *Staphylococcus aureus*, widely used as a biotechnological tool for the production of bioconjugates. Compared to a linear dimer, the cyclic dimer has a slightly increased thermal stability, is stable to exopeptidases, and retains high affinity, selective binding to HER2. The incorporation of a cysteine residue in the linker offers new opportunities for further functionalization, as demonstrated by the fluorescent labeling of the construct. This highly efficient enzyme-mediated strategy can readily be applied to the stabilization of different Affibody molecules, which are a class of engineered scaffold proteins emerging as viable alternatives to monoclonal antibodies for therapeutic applications.

## Figures and Tables

**Table 1:** Theoretical and observed molecular weights of proteins in this study.

Protein	Amino Acids	Theoretical MW (Da)	Observed MW (Da)
(Z_HER2_)_2:L_-Cys (linear Z_HER2_-dimer)	154	16,813	16,811 ^a^
(Z_HER2_)_2:L_ (linear iodoacetamide-blocked Z_HER2_-dimer)	154	16,870	16,868 ^a^
(Z_HER2_)_2:C_-Cys (cyclic Z_HER2_-dimer)	146	15,858	15,857 ^a^
(Z_HER2_)_2:C_-DL594 (cyclic DyLight 594-labeled Z_HER2_-dimer)	146	16,917	16,920 ^b^
(Z_HER2_)_2:C_ (cyclic iodoacetamide-blocked Z_HER2_-dimer)	146	15,915	15,915 ^b^

^a^ ESI-MS ^b^ MALDI-TOF MS, DL594 = DyLight 594 maleimide.

## Data Availability

The data generated during the study are available from the corresponding author upon reasonable request.

## Supplementary Materials

The following are available online, Figure S1: Representative chromatograms from the RP-HPLC purification of (ZHER2)2:L and (ZHER2)2:C; Figure S2: Representative chromatogram from the RP-HPLC purification of (ZHER2)2:C-DL594; Figure S3: Densitometry plots from SDS-PAGE gel analysis; Figure S4: ESI-MS spectrum of IMAC purified (ZHER2)2:L-Cys; Figure S5: ESI-MS spectrum of RP-HPLC purified (ZHER2)2:L; Figure S6: ESI-MS spectrum of IMAC purified (ZHER2)2:C-Cys; Figure S7: MALDI-TOF spectrum of RP-HPLC purified (ZHER2)2:C; and Figure S8: MALDI-TOF spectrum of RP-HPLC purified (Z_HER2_)_2:C_-DL594.

## Sample Availability

Samples of the compounds are not available from the authors.

## References

[1] Friedman M., Nordberg E., Höidén-Guthenberg I., Brismar H., Adams G., Nilsson F., Carlsson J., Ståhl S. (2007). Phage display selection of Affibody molecules with specific binding to the extracellular domain of the epidermal growth factor receptor. Protein Eng. Des. Sel..

[2] Orlova A., Magnusson M., Eriksson T.L., Nilsson M., Larsson B., Höidén-Guthenberg I., Widström C., Carlsson J., Tolmachev V., Ståhl S. (2006). Tumor Imaging Using a Picomolar Affinity HER2 Binding Affibody Molecule. Cancer Res..

[3] Kronqvist N., Malm M., Göstring L., Gunneriusson E., Nilsson M., Guthenberg I.H., Gedda L., Frejd F.Y., Ståhl S., Löfblom J. (2010). Combining phage and staphylococcal surface display for generation of ErbB3-specific Affibody molecules. Protein Eng. Des. Sel..

[4] Li J., Lundberg E., Vernet E., Larsson B., Höidén-Guthenberg I., Gräslund T. (2010). Selection of affibody molecules to the ligand binding site of the insulin-like growth factor-1 receptor. Biotechnol. Appl. Biochem..

[5] Honarvar H., Garousi J., Gunneriusson E., Höidén-Guthenberg I., Altai M. (2015). Imaging of CAIX-expressing xenografts in vivo using 99mTc-HEHEHE-ZCAIX:1 affibody molecule. Int. J. Oncol..

[6] Löfblom J., Feldwisch J., Tolmachev V., Carlsson J., Ståhl S., Frejd F. (2010). Affibody molecules: Engineered proteins for therapeutic, diagnostic and biotechnological applications. FEBS Lett..

[7] Ståhl S., Gräslund T., Eriksson Karlström A., Frejd F.Y., Nygren P.Å., Löfblom J. (2017). Affibody Molecules in Biotechnological and Med-ical Applications. Trends Biotechnol..

[8] Ekerljung L., Lennartsson J., Gedda L. (2012). The HER2-binding affibody molecule (Z(HER2:342))_2_ increases radiosensitivity in SKBR-3 cells. PLoS ONE.

[9] Conlan B.F., Gillon A.D., Craik D.J., Anderson M.A. (2010). Circular proteins and mechanisms of cyclization. Pept. Sci..

[10] Cobos E.S., Filimonov V.V., Gálvez A., Maqueda M., Valdivia E., Martinez J.C., Mateo P.L. (2001). AS-48: A circular protein with an extremely stable globular structure. FEBS Lett..

[11] Clark R.J., Craik D.J. (2010). ChemInform Abstract: Native Chemical Ligation Applied to the Synthesis and Bioengineering of Circular Peptides and Proteins. Pept. Sci..

[12] Bolscher J.G.M., Oudhoff M.J., Nazmi K., Antos J.M., Guimaraes C.P., Spooner E., Haney E.F., Vallejo J.J.G., Vogel H.J., Hof W.V. (2011). Sortase A as a tool for high-yield histatin cyclization. FASEB J..

[13] Iwai H., Plückthun A. (1999). Circular beta-lactamase: Stability enhancement by cyclizing the backbone. FEBS Lett..

[14] Antos J.M., Popp M.W.-L., Ernst R., Chew G.-L., Spooner E., Ploegh H.L. (2009). A Straight Path to Circular Proteins. J. Biol. Chem..

[15] Nguyen G.K., Kam A., Loo S., Jansson A.E., Pan L.X., Tam J.P. (2015). Butelase 1: A Versatile Ligase for Peptide and Protein Macrocy-clization. J. Am. Chem. Soc..

[16] Purkayastha A., Kang T.J. (2019). Stabilization of Proteins by Covalent Cyclization. Biotechnol. Bioprocess. Eng..

[17] Popp M.W.-L., Ploegh H.L. (2011). Making and Breaking Peptide Bonds: Protein Engineering Using Sortase. Angew. Chem. Int. Ed..

[18] Dai X.L., Boker A., Glebe U. (2019). Broadening the scope of sortagging. RSC Adv..

[19] Parthasarathy R., Subramanian S., Boder E.T. (2007). Sortase A as a novel molecular “stapler” for sequence-specific protein conjuga-tion. Bioconjug. Chem..

[20] Cheng X., Hong H., Zhou Z., Wu Z. (2018). Enzymatic On-Resin Peptide Cleavage and in Situ Cyclization One-Pot Strategy for the Synthesis of Cyclopeptide and Cyclotide. J. Org. Chem..

[21] Popp M.W., Dougan S.K., Chuang T.-Y., Spooner E., Ploegh H.L. (2011). Sortase-catalyzed transformations that improve the properties of cytokines. Proc. Natl. Acad. Sci. USA.

[22] Rasche N., Tonillo J., Rieker M., Becker S., Dorr B., Ter-Ovanesyan D., Betz U.A.K., Hock B., Kolmar H. (2016). PROLink—Single Step Circularization and Purification Procedure for the Generation of an Improved Variant of Human Growth Hormone. Bioconjug. Chem..

[23] Strijbis K., Spooner E., Ploegh H.L. (2012). Protein Ligation in Living Cells Using Sortase. Traffic.

[24] Ekblad T., Tolmachev V., Orlova A., Lendel C., Abrahmsén L., Eriksson Karlström A. (2009). Synthesis and chemoselective intramo-lecular crosslinking of a HER2-binding Affibody. Biopolymers.

[25] Järver P., Mikaelsson C., Karlström A.E. (2011). Chemical synthesis and evaluation of a backbone-cyclized minimized 2-helix Z-domain. J. Pept. Sci..

[26] Zhou F., Kroetsch A., Nguyen V.P., Huang X., Ogoke O., Parashurama N., Park S. (2019). High-Affinity Antibody Detection with a Bivalent Circularized Peptide Containing Antibody-Binding Domains. Biotechnol. J..

[27] Westerlund K., Honarvar H., Tolmachev V., Karlström A.E. (2015). Design, Preparation, and Characterization of PNA-Based Hybridization Probes for Affibody-Molecule-Mediated Pretargeting. Bioconjug. Chem..

[28] Kobashigawa Y., Kumeta H., Ogura K., Inagaki F. (2009). Attachment of an NMR-invisible solubility enhancement tag using a sortase-mediated protein ligation method. J. Biomol. NMR.

[29] Altai M., Westerlund K., Velletta J., Mitran B., Honarvar H., Karlström A.E. (2017). Evaluation of affibody molecule-based PNA-mediated radionuclide pretargeting: Development of an optimized conjugation protocol and 177Lu labeling. Nucl. Med. Biol..

[30] Waterhouse A., Bertoni M., Bienert S., Studer G., Tauriello G., Gumienny R., Heer F.T., de Beer T.A.P., Rempfer C., Bordoli L. (2018). SWISS-MODEL: Homology modelling of protein structures and complexes. Nucleic Acids Res..

[31] Scholtz J.M., Qian H., York E.J., Stewart J.M. (1991). Baldwin. Biopolymers.

[32] Eigenbrot C., Ultsch M., Dubnovitsky A., Abrahmsén L., Härd T. (2010). Structural basis for high-affinity HER2 receptor binding by an engineered protein. Proc. Natl. Acad. Sci. USA.

[33] Deis L.N., Pemble C.W., Qi Y., Hagarman A., Richardson D.C., Richardson J.S., Oas T.G. (2014). Multiscale conformational heterogeneity in staphylococcal protein a: Possible determi-nant of functional plasticity. Structure.

[34] Stiller C., Aghelpasand H., Frick T., Westerlund K., Ahmadian A., Karlström A.E. (2019). Fast and Efficient Fc-Specific Photoaffinity Labeling To Produce Antibody-DNA Conjugates. Bioconjug. Chem..

[35] Dincbas-Renqvist V., Lendel C., Dogan J., Wahlberg E., Härd T. (2004). Thermodynamics of Folding, Stabilization, and Binding in an Engineered Protein−Protein Complex. J. Am. Chem. Soc..

[36] Engfeldt T., Orlova A., Tran T., Bruskin A., Widström C., Karlström A.E., Tolmachev V. (2007). Imaging of HER2-expressing tumours using a synthetic Affibody molecule containing the 99mTc-chelating mercaptoacetyl-glycyl-glycyl-glycyl (MAG3) sequence. Eur. J. Nucl. Med. Mol. Imaging.

[37] Andersen J.T., Pehrson R., Tolmachev V., Daba M.B., Abrahmsén L., Ekblad C. (2011). Extending half-life by indirect targeting of the neonatal Fc receptor (FcRn) using a minimal albumin binding domain. J. Biol. Chem..

[38] Krasniqi A., Bialkowska M., Xavier C., Van der Jeught K., Muyldermans S., Devoogdt N., D’Huyvetter M. (2018). Pharmacokinetics of radiolabeled dimeric sdAbs constructs targeting human CD20. New Biotechnol..

[39] Tolmachev V., Orlova A., Pehrson R., Galli J., Baastrup B., Andersson K., Sandström M., Rosik D., Carlsson J., Lundqvist H. (2007). Radionuclide therapy of HER2-positive microxenografts using a 177Lu-labeled HER2-specific Af-fibody molecule. Cancer Res..

[40] Altai M., Liu H., Ding H., Mitran B., Edqvist P.-H., Tolmachev V., Orlova A., Gräslund T. (2018). Affibody-derived drug conjugates: Potent cytotoxic molecules for treatment of HER2 over-expressing tumors. J. Control. Release.

[41] Nam J.M., Jeon K.-H., Kwon H., Lee E., Jun K.-Y., Jin Y.B., Lee Y.-S., Na Y., Kwon Y. (2013). Dithiiranylmethyloxy azaxanthone shows potent anti-tumor activity via suppression of HER2 expression and HER2-mediated signals in HER2-overexpressing breast cancer cells. Eur. J. Pharm. Sci..

