# Supplementary material for: Stability Enhancement of a Dimeric HER2-Specific Affibody Molecule through Sortase A-Catalyzed Head-to-Tail Cyclization
Source: Molecules. 2021 May 12;26(10):2874. doi: 10.3390/molecules26102874 (PMC8150554; doi:10.3390/molecules26102874)
Supplement: Supplementary file 1 [file molecules-26-02874-s001.zip › molecules-1188641-supplementary.pdf]

Supplementary Information

Stability enhancement of a dimeric HER2-specific Affibody molecule through sortase A- catalyzed head-to-tail cyclization

Kristina Westerlund, Anders Myrhammar, Hanna Tano, Maxime Gestin and Amelie Eriksson Karlström

Department of Protein Science
School of Engineering Sciences in Chemistry, Biotechnology and Health
KTH Royal Institute of Technology
AlbaNova University Center
SE-10691 Stockholm, Sweden

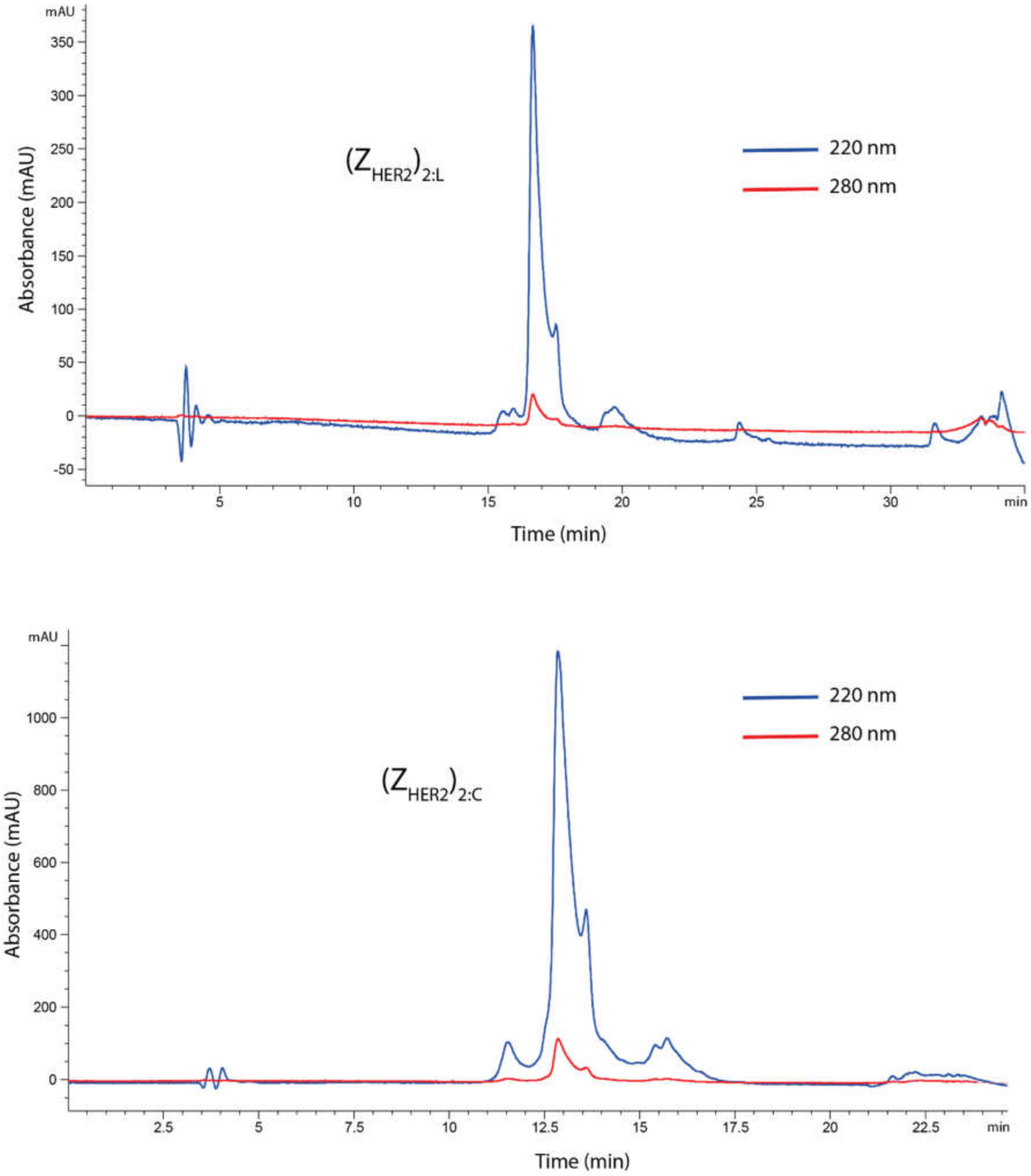

Figure S1. Representative chromatograms from the RP-HPLC purification of A) $(Z_{HER2})_{2:L}$ and B) $(Z_{HER2})_{2:C}$. The purification was done on a Zorbax C18 column (300SB-C18, 9.4 × 250 mm, 5 µm particle size; Agilent, Santa Clara, CA, USA) using a flow rate of 3 ml min^{-1}. The gradient used to purify $(Z_{HER2})_{2:L}$ was 25 to 55 % B over 30 min (solvent A = 0.1% TFA-H_2O, solvent B = 0.1% TFA-CH_3CN), and the gradient used to purify $(Z_{HER2})_{2:C}$ was 35 to 45% B over 20 min. The main peak absorbing at 220 nm in A was collected in two different fractions but when MALDI-TOF investigation revealed that both fractions contained pure $(Z_{HER2})_{2:L}$ the fractions were combined. The same procedure was done with $(Z_{HER2})_{2:C}$ in B.

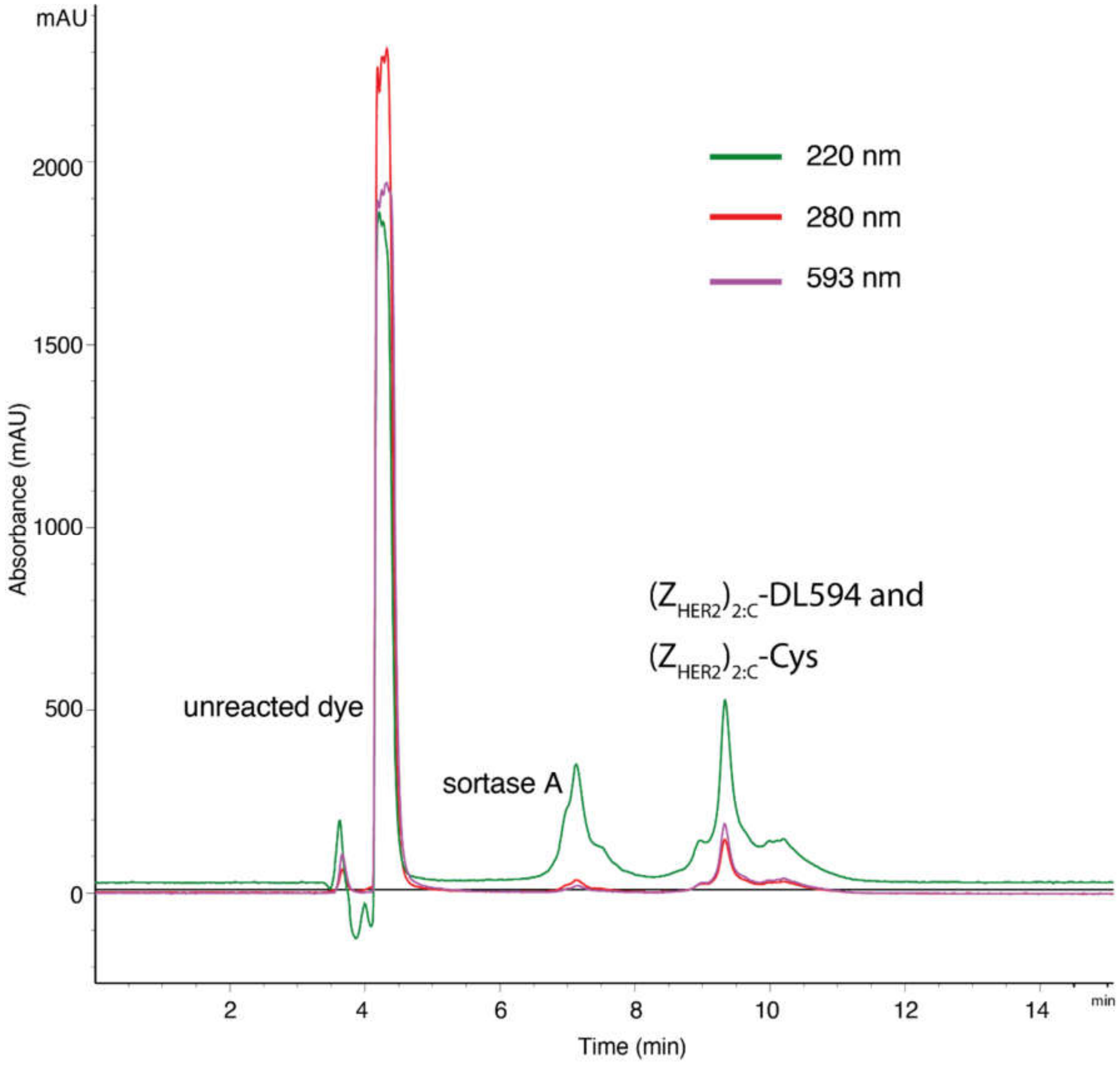

Figure S2. Representative chromatograms from the RP-HPLC purification of $(Z_{HER2})_{2:C}$-DL594. The purification was done on a Zorbax C18 column (300SB-C18, 9.4 × 250 mm, 5 µm particle size; Agilent, Santa Clara, CA, USA) using a flow rate of 3 ml min^{-1}. The gradient used to purify $(Z_{HER2})_{2:C}$-DL594 was 30 to 45% B over 15 min. The identities of the proteins in peaks labeled in the chromatogram was investigated using MALDI-TOF MS. $(Z_{HER2})_{2:C}$-DL594 coeluted with the unlabeled cyclic Z_{HER2}-dimer in RP-HPLC, for the MALDI-TOF spectra of the peak containing $(Z_{HER2})_{2:C}$-DL594 and $(Z_{HER2})_{2:C}$-Cys see Figure S7.

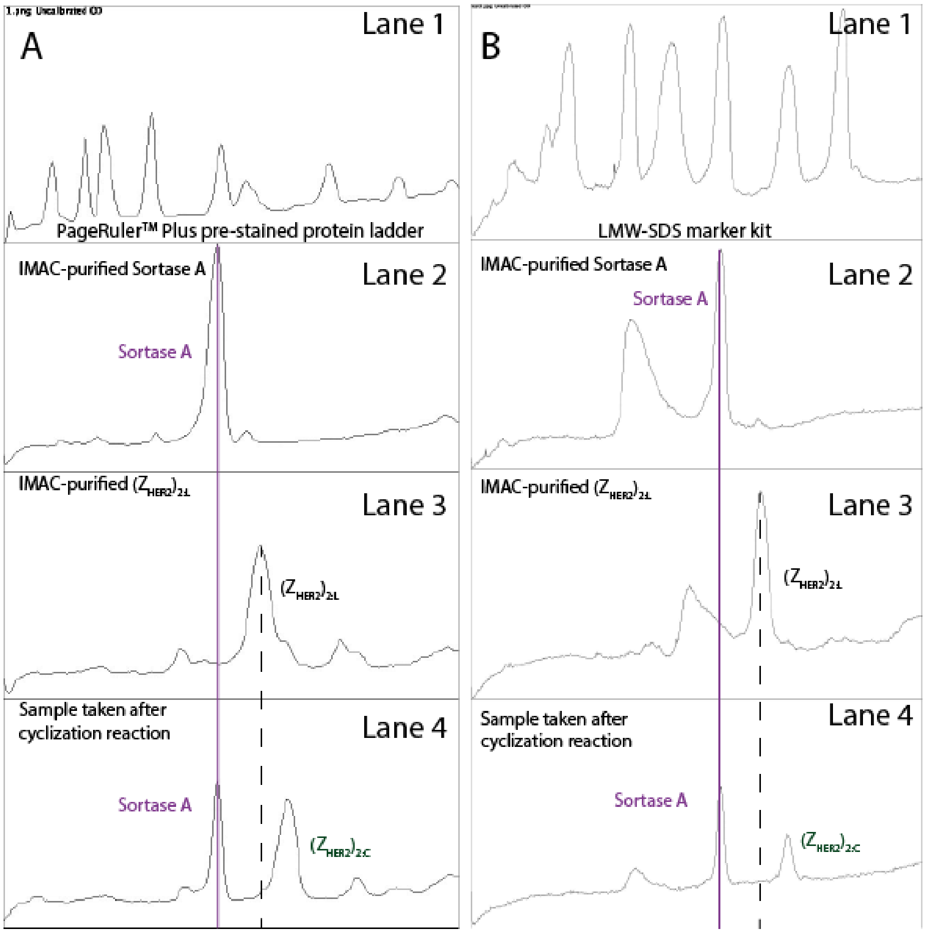

Figure S3. Densitometry plots in A (left) and B (right) generated from SDS-PAGE gel analysis of two different cyclization reactions. The SDS-PAGE gel used to generate the plot in A can be seen in Figure 3 in the main text. From lane 4 in each gel, the reaction yield of the sortase A-catalyzed cyclization reaction of $(Z_{HER2})_{2:C}$ was estimated by comparing the integrated areas under the $(Z_{HER2})_{2:L}$ and $(Z_{HER2})_{2:C}$ peaks. The yield of the cyclization reaction was estimated to be in the 99.5-95.5% range depending on gel and analysis method, but was complicated by the similarity in mobility between $(Z_{HER2})_{2:L}$ and $(Z_{HER2})_{2:C}$. In the main text the reaction yield is given as >95%.

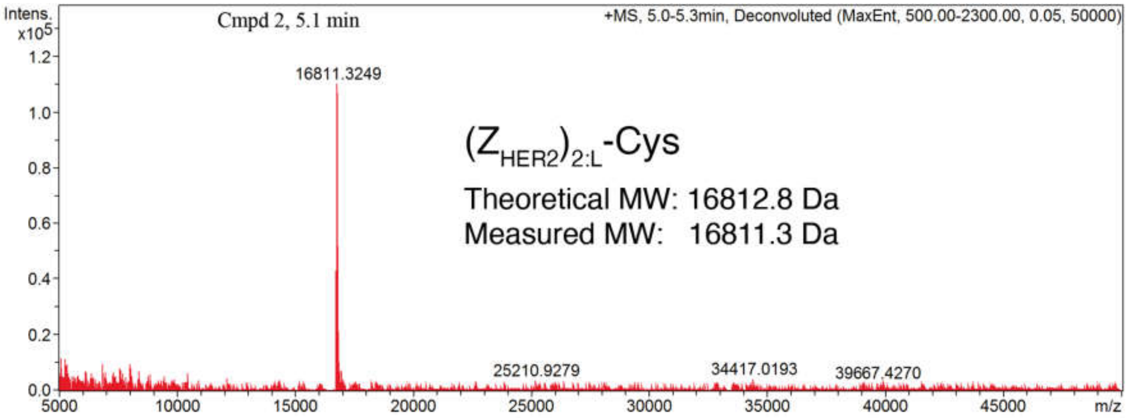

Figure S4. ESI-MS spectrum of IMAC purified $(Z_{HER2})_{2:L}$-Cys. The analysis was performed using a Thermo Ultimate 3000 LC system (Thermo Fisher Scientific, Waltham, MA, USA) and a Bruker Impact II mass spectrometer (Bruker Daltonics, Billerica, MA, USA). The observed molecular weight is 16811 Da, and the theoretical molecular weight is 16813 Da.

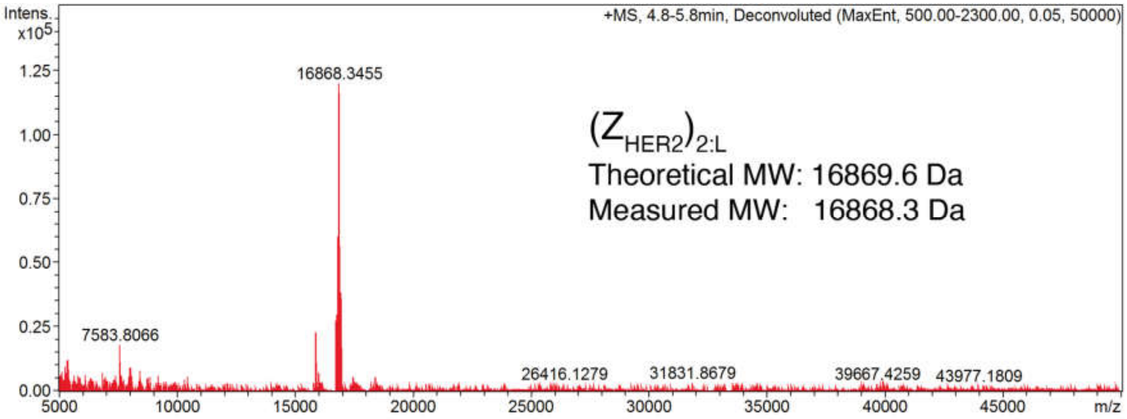

Figure S5. ESI-MS spectrum of RP-HPLC purified (Z_{HER2})$_{2:L}$. The analysis was performed using a Thermo Ultimate 3000 LC system (Thermo Fisher Scientific, Waltham, MA, USA) and a Bruker Impact II mass spectrometer (Bruker Daltonics, Biller-ica, MA, USA). The observed molecular weight is 16868 Da, and the theoretical molecular weight is 16870 Da.

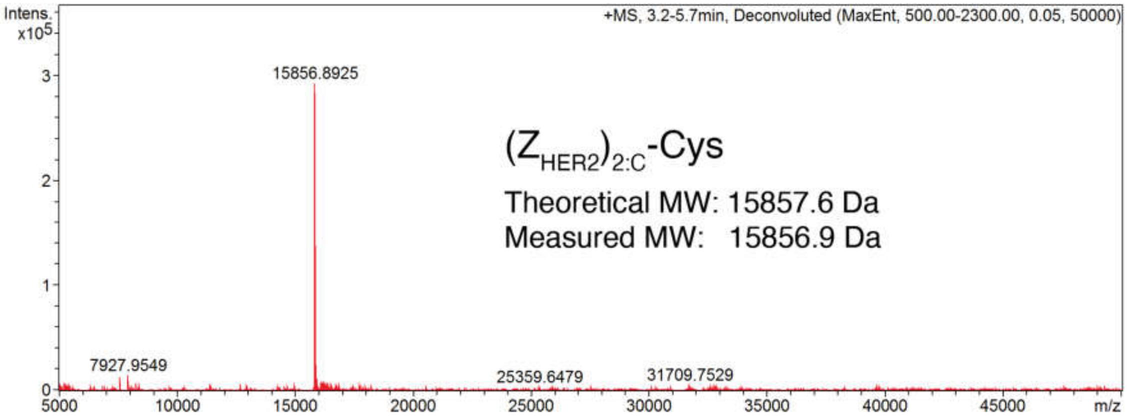

Figure S6. ESI-MS spectrum of IMAC purified (Z_{HER2})$_{2:C}$-Cys. The analysis was performed using a Thermo Ultimate 3000 LC system (Thermo Fisher Scientific, Waltham, MA, USA) and a Bruker Impact II mass spectrometer (Bruker Daltonics, Billerica, MA, USA). The observed molecular weight is 15857 Da, and the theoretical molecular weight is 15858 Da.

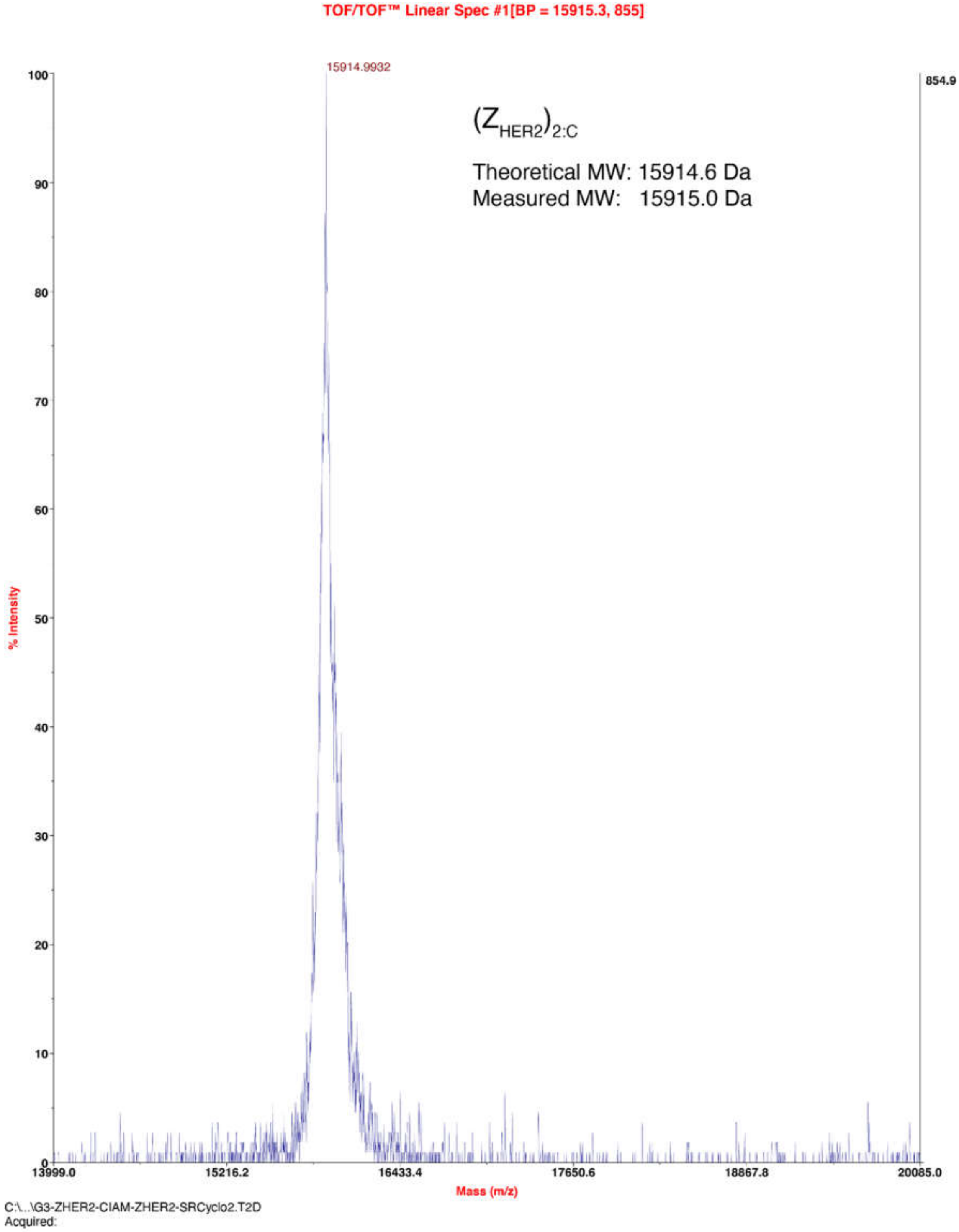

Figure S7. MALDI-TOF spectrum of RP-HPLC purified $(Z_{HER2})_{2:C}$. The analysis was performed using a 4800 MALDI-TOF/TOF mass spectrometer (AB SCIEX, Framingham, MA, USA). The observed molecular weight is 15915 Da, and the theoretical molecular weight is 15915 Da.

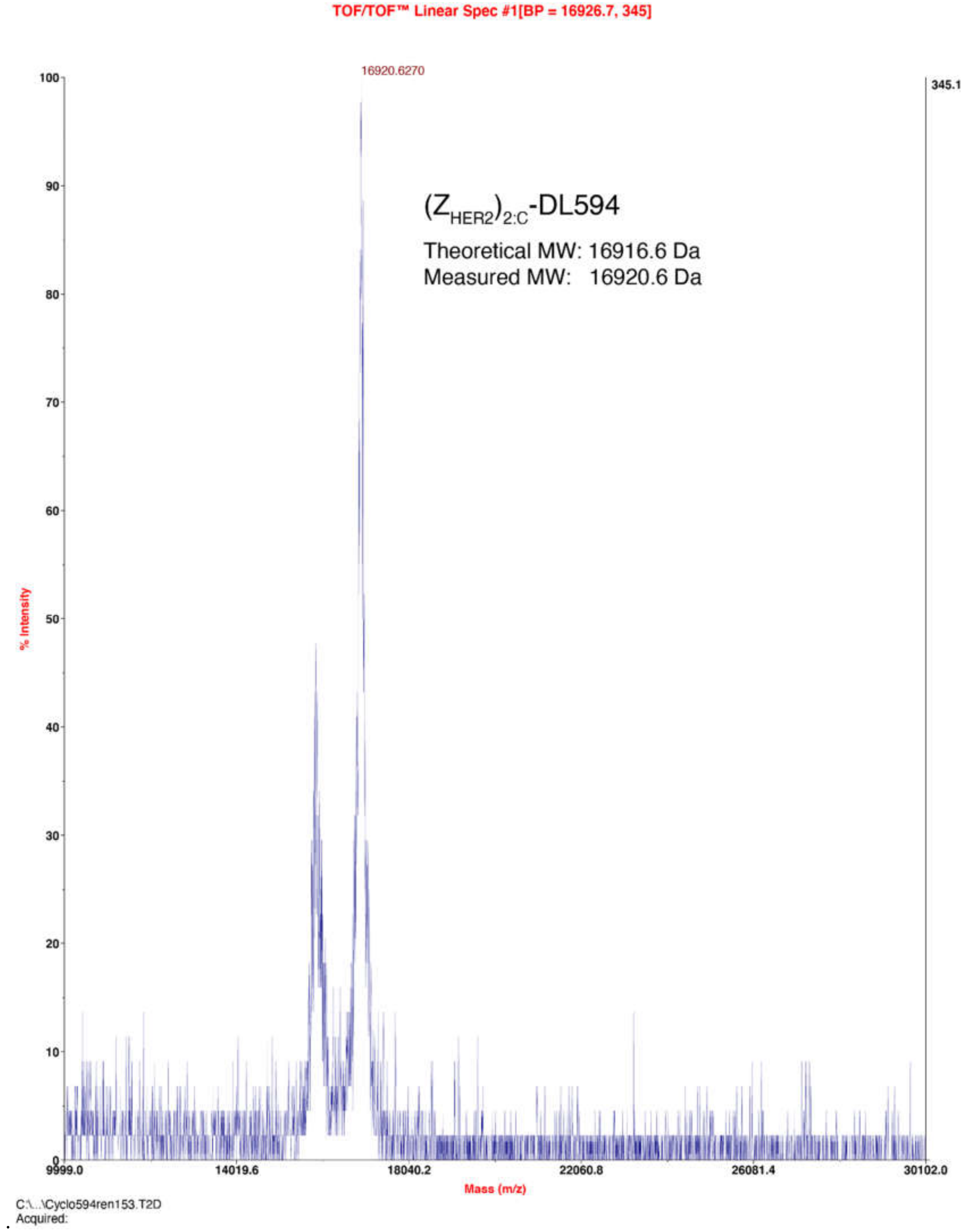

Figure S8. MALDI-TOF spectrum of RP-HPLC purified $(Z_{HER2})_{2:C}$-DL594. The analysis was performed using a 4800 MALDI-TOF/TOF mass spectrometer (AB SCIEX, Framingham, MA, USA). The observed molecular weight of the main peak is 16920 Da, and the theoretical molecular weight of $(Z_{HER2})_{2:C}$-DL594 is 16917 Da. The smaller peak at around 16000 Da is probably unlabeled $(Z_{HER2})_{2:C}$-Cys, with a theoretical molecular weight of 15858 Da.
